# Evaluation of semi-preloaded intraocular lens delivery system

**Published:** 2019

**Authors:** Rajesh Rajesh

**Affiliations:** * Department of Ophthalmology, Vasantrao Naik Government Medical, College, Maharashtra, India

**Keywords:** preloaded intraocular lens, phacoemulsification, intraocular lenses, CT Lucia

## Abstract

**Purpose:** In this study, we aimed to evaluate the single surgeon experience of semi-preloaded intraocular lens (IOL) delivery system.

**Methods:** Phacoemulsification was performed under topical anesthesia by temporal clear corneal incision. CT Lucia hydrophobic IOL was injected through semi- preloaded IOL system in the capsular bag. Two hundred patients (200 eyes) were included in the study. The main outcome measures were ease of implantation, intraoperative and postoperative complications, postoperative centration, and visual acuity. Data on successful implantation and complications were collected prospectively.

**Results:** Correct IOL delivery was achieved in 193 out of 200 patients (96.5%). Four patients (2%) required intrawound rotation of the injector to place the leading haptic in the capsular bag. Two patients (1%) had anteroposterior rotation of the IOL and one patient (0.5%) had total posterior rotation of IOL. Other problems noted were trapped trailing haptic (n=2,1%), improper loading of IOL (n=3,1.5%) and stretch marks on the optic of IOL (n=4,2%). None of the patients had iris trauma or posterior capsular rupture during the implantation and manipulation of the IOL.

The mean incision size after completion of implantation of IOL was 2.82 mm (+ 0.02), which achieved sutureless closure. None of the patients developed postoperative infection.

**Conclusion:** Implantation of CT Lucia 601 PY IOL with the semi preloaded system led to minor complications and gave satisfactory visual results.

## Introduction

Modern phacoemulsification system allows the implantation of foldable intraocular lens (IOL) through a small incision. Foldable IOLs are recognized worldwide for their advantages, which include reduction in surgically induced astigmatism, reduced forceps use in handling polymethylmethaacrylate rigid IOL and reduced bacterial entry into the eye due to no contact between IOL and operative field. Various injector systems employed for the injection of IOL include manual folding of IOL using forceps and unfolder cartridge mounted on either reusable metallic or disposable injector. These injector systems encompass handling of IOL by forceps. Metallic injectors require maintenance, i.e., cleaning and autoclaving before each use. Issues related with these injector systems include forceps-induced scratch mark on the optics of IOL [**1**], irregularities on the surface of the optic due to compression of IOL during packaging [**2**], stretch mark on the posterior surface of IOL during injection [**3**], cartridge shaft deformity leading to protrusion of IOL through cartridge shaft [**4**] and surgeon’s error in holding and folding of IOL leading to reversal of optic [**1**,**5**]. To solve these concerns, preloaded IOLs have been developed and its recognized benefits include reduced surgical time and uniformity in loading of IOL [**6**]. 

In the Asian part of the world, there is a rise in the use of foldable IOLs and preloaded IOLs have been recently introduced on the markets. However, no study on preloaded IOL system exists for Asian population.

In this study, we have observed delivery characteristics and safety features of implantation of the newly introduced semi-preloaded IOL system by Zeiss. 

## Methods

This prospective observational study was performed in the tertiary eye care center, which is situated in the central province of India. The study included those patients with diagnosis of cataract who had applied to the hospital from January to October 2017. The hospital ethical committee approved this study. 

A total of 200 patients (102 males and 98 females) implanted with semi-preloaded IOL (CT Lucia 601 PY, Zeiss Optics, Carl Zeiss Meditec AG, Germany) were included in the study. All patients provided a written informed consent, and the study was conducted in accordance with the tenets of the Declaration of Helsinki. 

Routine preoperative examination was performed and the Lens Opacities Classification System III was used for nucleus grading [**7**]. 

Preoperative mydriasis was achieved using phenylephrine 5% and tropicamide 0.8% eye drops. A single experienced surgeon operated on the patients who were instilled with topical anesthetic drops (Proparacain HCL 0.5%) thrice at an interval of 5 min. A side port incision was fashioned on left side. Viscoelastic material (2% Hydroxypropyl methylcellulose, Appavisc, Appasamy Ocular Devices, Puducherry, India) was injected to facilitate creation of 2.8 mm clear corneal temporal incision. Capsulorhexis was achieved with the help of utrata forceps. Hydrodissection was performed and nucleus was freed by dialing. A standard quick chop was applied for endocapsular phacoemulsification (Galaxy Phacoemulsifier, Appasamy Ocular Devices, Puducherry, India). Cortical aspiration was completed by irrigation/ aspiration probe. Anterior chamber was filled with viscoelastic.

The surgeon had the preloaded IOL along with injector system in his left hand (CT Lucia 601 PY, Zeiss Optics, Carl Zeiss Meditec AG, Germany, 6 mm optic, 13 mm overall diameter, hydrophobic and heparin coated). The tip of the cartridge was faced to the left. According to the manufacturer’s instructions, the flanges of the cartridge were closed so that the protecting cover could be released. A click sound indicated a proper closure of the cartridge. Viscoelastic was injected through the front portion of the cartridge up to the pusher in the injector. The surgeon held the injector in his right hand and placed the tip of the cartridge in the clear corneal incision with the bevel opening in the anterior chamber. The delivery of the IOL was achieved with a further advancement of the leading haptic into the capsular bag. The trailing haptic was then dialed into the bag with a second manipulating instrument to achieve a well-centered IOL position. After each surgery, incision size was measured before and after the implantation of IOL and the surgeon noted down the loading characteristics of the IOL. 

**Fig. 1:**
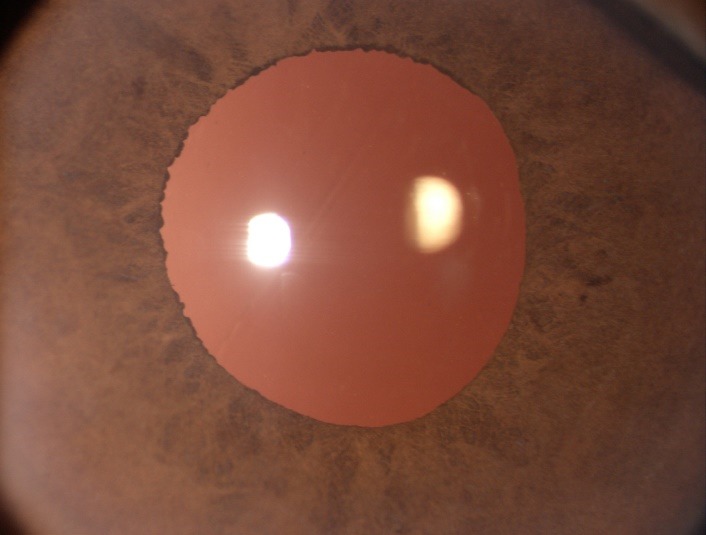
Postoperative day one follow up

**Fig. 2:**
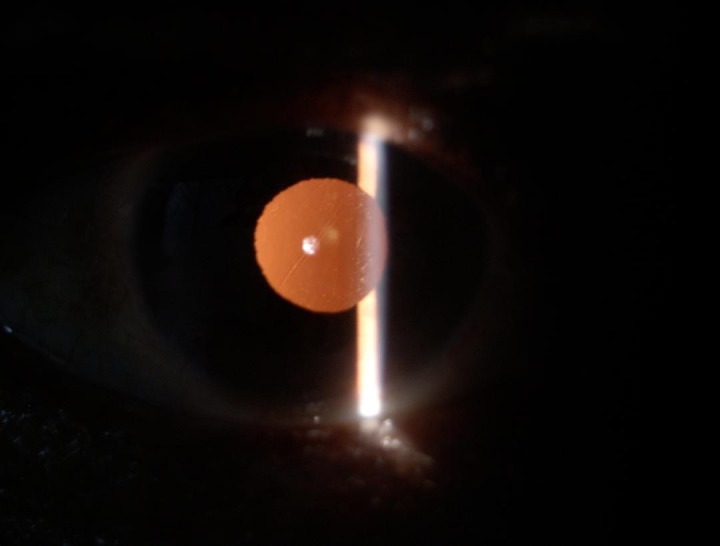
Postoperative 6 weeks follow up

Postoperative follow up was done on day one (**Fig. 1**), after one week, one month and at six months (**Fig. 2**).

## Results 

The mean age of the participants at the time of surgery was 69.42 years (+12.05). The IOL powers of the inserted CT Lucia 601 PY ranged from 17 to 28 diopters. One hundred and ninety three patients (96.5%) did not require additional manipulation in the anterior chamber to place IOL in the capsular bag. Four patients (2%) required rotation of the injector (varying from 10–90°) in the wound to place the leading haptic in the capsular bag. Two patients (1%) had anteroposterior rotation of the IOL and one patient (0.5%) had total posterior rotation of IOL, wherein posterior surface of the IOL was facing anteriorly. Position of IOL was corrected subsequently and all the patients had uneventful delivery of leading and trailing haptic in the bag. In two patients (1%), the trailing haptic was trapped between the syringe plunger and the nozzle. The manipulation of the plunger freed the haptic exterior to the wound, which was placed in the capsular bag with dialer. None of the patients had iris trauma or posterior capsular rupture during implantation and manipulation of the IOL. Optic haptic adhesion was not seen in any case. After the loading of IOL in the cartridge, lens optic along with its folded haptics was clearly seen. On three occasions (1.5%), the surgeon had doubts about improper loading of the IOL. Preloaded IOL was removed from the cartridge, and another injector for hydrophilic IOL injection was taken and the procedure was completed. Stretch mark on the optics of IOL was seen in four patients (2%), who had an IOL power between 26–28 D. However, postoperative follow up was uneventful in all the cases. The final position of all the IOLs was centered in the capsular bag. The mean incision size after completion of phacoemulsification and implantation of IOL was 2.82 mm (+ 0.02), which achieved sutureless closure.

One hundred and ninety four patients (97%) achieved best-corrected visual acuity of 20/ 20 at six months follow up. Four of the remaining six patients had visual acuity of 20/ 80 and had age-related macular degeneration, and two patients (20/60) had partial optic atrophy. None of the patients developed postoperative infection. 

## Discussion

Cataract surgery is the most commonly performed ocular surgery throughout the world. Phacoemulsification with the implantation of a foldable IOL has become a standard method for cataract treatment. Reduction in the size of the incision improves the delivery system of IOL. Introduction of preloaded IOL delivery has added safety to the insertion of IOL through the clear corneal incision. It ensures human error free delivery of IOL. Our experience with newly developed preloaded IOL injection system showed that 193 out of 200 patients (96.5%) did not require additional manipulation in the anterior chamber to place the IOL in the capsular bag during its delivery. This observation is better than the study by Ong et al. on AcrySert preloaded IOL delivery system (55% required additional manipulation) [**8**]. This proves the safety of preloaded injector system for IOL implantation used in the study. 

An attempt was made to place the leading haptic in the capsular bag during injection to avoid the opening of IOL in the anterior chamber. In that endeavor, four (2%) patients needed intrawound rotation of the injector, which was associated with IOL rotation in the anterior chamber. Total reversal of optic, in which anterior surface of IOL was facing posteriorly, occurred in one case. Such reversal was due to the improper loading and holding of IOL in unfolder system [**5**]. Ong et al. termed such cases as “flipped” IOL position [**8**]. We could correct the IOL position by introducing dialers through the side port and main incision, which occurred during the learning stage of the implantation. 

An ideal IOL injector system should have minimal or no rotation of injector system during implantation to avoid damage to the architecture of the corneal wound. Damage to the wound construction requires suture at the incision site. None of the patients required suture at the incision site. Incision size remained unchanged after the implantation of IOL. 

We call this system as semi-preloaded as the part of lens is exposed to the operation theater environment after the removal of lens protecting cover. In preloaded system, the lens is preloaded in the injector and ready for injection. 

Various investigators have noted haptic entrapment within the cartridge during the use of unfolder system and preloaded IOL delivery system [**8**-**11**]. In our study, two patients had trapped trailing haptic, which occurred due to injector system plunger along with its sleeve overriding trailing haptic optic junction. Withdrawing the plunger along with its sleeve rectified the situation. The sleeve being loosely attached to the proximal end of the plunger has a tendency to override stiff trailing haptic optic junction. 

On three occasions, the surgeon was unsure about the loading of IOL. The IOL was reloaded in a new injector system available for hydrophilic IOL and the procedure was completed. It is important to observe the passage of IOL through the cartridge during injection to avoid problems related to its improper loading. IOL was visible during the passage through the cartridge in all cases. 

Stretch lines on central and peripheral part of IOL optic were seen in four cases. All four patients had IOL power in the range of 26–28 diopters. There was a concern about the drop in the visual acuity in these patients who had stretch lines on the central part of the optic. However, postoperative follow up did not show stretch marks nor did it affect the visual outcome. The thickness of IOL depends on its power. In spite of putting the adequate viscoelastic agent in the cartridge, the increase in IOL thickness increases the chance of optic rubbing the wall of cartridge. 

Literature has reported delayed onset postoperative infection after the implantation of preloaded IOL [**12**,**13**]. However, none of the patients developed postoperative infection in the follow up period of six months.

Limitations of this study include the absence of a control group (non-preloaded IOL system) and the involvement of a single surgeon, which omits the comparison of techniques of implantation of IOL with different surgeons.

Despite the difficulties mentioned in the case series, none of the patients in the study suffered complications affecting the final visual acuity. 

**Acknowledgement**

Nil.

**Source of funding**

Nil. 

## References

[1] Mencucci R, Dei R, Danielli D, Susini M, Menchini U (2004). Folding procedure for acrylic intraocular lenses. J Cataract Refract Surg.

[2] Nguyen DQ, Saleh TA, Pandey SK, Bates AK (2006). Irregularities on the surface of single-piece AcrySof SA60AT intraocular lenses. J Cataract Refract Surg.

[3] Faschinger CW (2001). Surface abnormalities on hydrophilic acrylic intraocular lenses implanted by an injector. J Cataract Refract Surg.

[4] Joshi RS (2013). Descemet's tear due to injector cartridge tip deformity: Cartridge shaft deformity. Indian J Ophthalmol.

[5] Rao SK, Leung AT, Lam DS (2000). Padmanabhan P. In situ tumbling of the AcrySof intraocular lens. J Cataract Refract Surg.

[6] Shimizu K, Kobayashi K, Takayama S, Zhaobin G (2008). Preloaded injector for intraocular lens implantation without the use of ophthalmic viscosurgical devices. J Cataract Refract Surg.

[7] Chylack LT Jr, Wolfe JK, Singer DM, Leske MC, Bullimore MA, Bailey IL (1993). The lens opacities classification system III. Arch Ophthalmol.

[8] Ong HS, Subash M, Sandhu A, Wilkins MR (2013). Intraocular lens delivery characteristics of the preloaded AcrySof IQ SN60WS/AcrySert injectable lens system. Am J Ophthalmol.

[9] Ng DT, Francis IC, Schumacher RS, Alexander SL (2001). Prospective study of 1 surgeon's experience with 115 cases using the Unfolder lens injection system. J Cataract Refract Surg.

[10] Stefaniu I, Nita N, Lazar S, Dragan I, Barlea G, Balan L, Hagima N (2003). Acrylic IOL implantation with the Monarch II injector. Oftalmologia (Bucharest, Romania: 1990).

[11] Barakova D, Kuchynka P, Cihelkova I (2002). Implantation of the AcrySof MA30BA lens using the Monarch system. Ceska a slovenska oftalmologie: casopis Ceske oftalmologicke spolecnosti a Slovenske oftalmologicke spolecnosti.

[12] Hayashi Y, Eguchi H, Miyamoto T, Inoue M, Mitamura Y (2012). A Case of Delayed-Onset Propionibacterium acnes Endophthalmitis after Cataract Surgery with Implantation of a Preloaded Intraocular Lens. Case Rep Ophthalmol.

[13] Kokuzawa S, Suemori S, Mochizuki K, Hirose Y, Yaguchi T (2013). Aspergillus TubingenesisEndophthalmitis after Cataract Surgery with Implantation of Preloaded Intraocular Lens. Semin Ophthalmol.

